# Prolonged exposure of mouse and human podocytes to insulin induces insulin resistance through lysosomal and proteasomal degradation of the insulin receptor

**DOI:** 10.1007/s00125-017-4394-0

**Published:** 2017-08-29

**Authors:** Abigail C. Lay, Jenny A. Hurcombe, Virginie M. S. Betin, Fern Barrington, Ruth Rollason, Lan Ni, Lawrence Gillam, Grace M. E. Pearson, Mette V. Østergaard, Hellyeh Hamidi, Rachel Lennon, Gavin I. Welsh, Richard J. M. Coward

**Affiliations:** 1 Bristol Renal, Bristol Medical School, University of Bristol, Whitson Street, Bristol, BS1 3NY UK; 2 Global Research, Novo Nordisk A/S, Måløv, Denmark; 3 Wellcome Trust Centre for Cell-Matrix Research, Division of Cell Matrix Biology and Regenerative Medicine, School of Biology, Faculty of Biology, Medicine and Health, Manchester Academic Health Science Centre, University of Manchester, Manchester, UK

**Keywords:** Albuminuria, Diabetic nephropathy, Genetic background, Insulin resistance, Kidney injury

## Abstract

**Aims/hypothesis:**

Podocytes are insulin**-**responsive cells of the glomerular filtration barrier and are key in preventing albuminuria, a hallmark feature of diabetic nephropathy. While there is evidence that a loss of insulin signalling to podocytes is detrimental, the molecular mechanisms underpinning the development of podocyte insulin resistance in diabetes remain unclear. Thus, we aimed to further investigate podocyte insulin responses early in the context of diabetic nephropathy.

**Methods:**

Conditionally immortalised human and mouse podocyte cell lines and glomeruli isolated from *db/db* DBA/2J mice were studied. Podocyte insulin responses were investigated with western blotting, cellular glucose uptake assays and automated fluorescent imaging of the actin cytoskeleton. Quantitative (q)RT-PCR was employed to investigate changes in mRNA. Human cell lines stably overproducing the insulin receptor (IR) and nephrin were also generated, using lentiviral constructs.

**Results:**

Podocytes exposed to a diabetic environment (high glucose, high insulin and the proinflammatory cytokines TNF-α and IL-6) become insulin resistant with respect to glucose uptake and activation of phosphoinositide 3-kinase (PI3K) and mitogen-activated protein kinase (MAPK) signalling. These podocytes lose expression of the IR as a direct consequence of prolonged exposure to high insulin concentrations, which causes an increase in IR protein degradation via a proteasome-dependent and bafilomycin-sensitive pathway. Reintroducing the IR into insulin-resistant human podocytes rescues upstream phosphorylation events, but not glucose uptake. Stable expression of nephrin is also required for the insulin-stimulated glucose uptake response in podocytes and for efficient insulin-stimulated remodelling of the actin cytoskeleton.

**Conclusions/interpretation:**

Together**,** these results suggest that IR degradation, caused by high levels of insulin, drives early podocyte insulin resistance**,** and that both the IR and nephrin are required for full insulin sensitivity of this cell. This could be highly relevant for the development of nephropathy in individuals with type 2 diabetes, who are commonly hyperinsulinaemic in the early phases of their disease.

**Electronic supplementary material:**

The online version of this article (doi:10.1007/s00125-017-4394-0) contains peer-reviewed but unedited supplementary material, which is available to authorised users.

## Introduction

Diabetic nephropathy, in which albuminuria is an early manifestation, occurs in approximately one third of diabetic individuals and is the leading cause of end-stage renal failure worldwide [1]. It is well recognised that insulin resistance plays a major role in the pathogenesis of both type 1 and type 2 diabetes [2, 3], including a role in the development of renal complications. Interestingly, in addition to being associated with albuminuria and nephropathy in both type 1 [4, 5] and type 2 [6] diabetes, insulin resistance is also associated with the development of albuminuria in non-diabetic individuals [7]. Renal disease is also common among people with severe forms of genetic insulin resistance [8].

A range of circulating factors are dysregulated early in the development of systemic insulin resistance, as either a cause or a consequence of disrupted cellular insulin signalling. Among these factors, elevated inflammatory cytokines, hyperglycaemia and hyperinsulinaemia are particularly prominent [9]. Although the negative regulation of insulin signalling is relatively well characterised in classical insulin-responsive tissues, such as skeletal muscle and liver, the impairment of insulin action does not occur uniformly throughout all insulin-responsive tissues, and the effects of factors associated with insulin resistance are often tissue-specific [10].

Podocytes are specialised epithelial cells lining the urinary side of the glomerular filtration barrier in the kidney, essential in the maintenance of glomerular function. Podocyte loss or disturbance is linked to the development of albuminuria and occurs early in the progression of diabetic nephropathy [11–14].

We previously demonstrated that podocytes are insulin-responsive cells in vitro [15], and the specific deletion of the podocyte insulin receptor (IR) in vivo disrupts glomerular function, causing features reminiscent of diabetic nephropathy, independent of hyperglycaemia [16]. Furthermore, a podocyte-specific reduction in IR production has been found to exacerbate albuminuria in a mouse model of diabetic nephropathy [17], further highlighting the importance of podocyte IR signalling in disease.

There is also increasing evidence that podocyte insulin responses may be negatively regulated in situations of systemic insulin resistance [18, 19]. In particular, the finding that podocytes isolated from *db/db* mice display reduced insulin-stimulated Akt phosphorylation may suggest that circulating factors, associated with type 2 diabetes, have the capacity to disrupt podocyte insulin responses early in the course of glomerular disease [20]. Despite this, relatively little is still known about the specific factors that regulate podocyte insulin responses.

This study aimed to investigate how factors associated with systemic insulin resistance influence podocyte insulin signalling and, consequently, the development of renal disease in situations of insulin resistance, including diabetic nephropathy.

## Methods

### Animals

All animal experiments and procedures were approved by the UK Home Office in accordance with the Animals (Scientific Procedures) Act 1986, and the Guide for the Care and Use of Laboratory Animals was followed during experiments. Heterozygous *db/wt* DBA/2J (D2.BKS(D)-*Lepr* ^*db*^/J) mice were purchased from the Jackson Laboratory (Bar Harbor, ME, USA). Female and male *db/db* mice were bred in house, as described [21]. Glomeruli were isolated after perfusion with dynabeads (Thermo Fisher, Paisley, UK). Further details are available in the electronic supplementary material (ESM).

### Generation of podocyte cell lines from *db/db* and wild-type mice

Podocytes were isolated from perfused glomeruli from a male *db/db* DBA/2J and male wild-type (WT) DBA/2J littermate control mouse at 12 weeks of age. These podocytes were conditionally immortalised with temperature-sensitive SV40 transfection as previously described [22, 23].

### Cell culture

Conditionally immortalised human [24] and mouse [22] podocytes were maintained in RPMI-1640 containing l-glutamine and NaHCO_3_, supplemented with 10% FBS (Sigma Aldrich, Gillingham, UK). Cells were studied after 12–14 days differentiation at 37°C and were free of *Mycoplasma* infection.

### Cell treatments

To mimic a diabetic environment in vitro*,* podocytes were grown in the presence of 100 nmol/l insulin (Tocris, Bristol, UK), 25 mmol/l glucose (Sigma), 1 ng/ml TNF-α and 1 ng/ml IL-6 (R&D systems, Abingdon, UK). d-Mannitol (Sigma) was used as a control for osmotic pressure in these assays. For initial chronic insulin exposure, podocytes were incubated with insulin at 10 nmol/l and 100 nmol/l for 10 days. Although supraphysiological (as physiological hyperinsulinaemia is typically within the range 1000–2000 pmol/l, occurring over an extended period of months or years), this is consistent with numerous in vitro studies of other cell types [25–30]. For short-term insulin stimulation, culture medium was replaced with serum- and insulin-free RPMI-1640 for 2–4 h, and podocytes were re-challenged with insulin at 10 or 100 nmol/l for 10 min. For inhibition of proteasomal and lysosomal degradation, podocytes were treated with 10 μmol/l MG-132 (Sigma) or 50 nmol/l bafilomycin (Tocris), respectively, for 8 h.

### Lentiviral transfection of podocytes

Human IR (NM_000208.2) was subcloned into pLenti-TetCMV(IR)-Rsv(RFP-Bsd) expression vector (Gentarget, San Diego, CA, USA). The human nephrin expression plasmid (pWPXL-Nephrin-FLAG) was a gift from R. Lennon (University of Manchester). Expression vectors were transfected into Lenti-X 293T cells (Clontech/Takara Bio Europe SAS, Saint-Germain-en-Laye, France), together with packaging vectors pMD.2G (Addgene no. 12259) and psPAX2 (Addgene no. 12260), both gifts from D. Trono (École polytechnique fédérale de Lausanne), as previously reported [31]. Lentiviral particles were purified from the cell supernatant fraction, and immortalised podocytes were transduced overnight in the presence of polybrene. IR-containing podocytes were selected using blasticidin.

### Glucose uptake assays

Insulin-stimulated glucose uptake into podocytes was measured as previously described [15]. Briefly, cells were serum-starved before incubation with a modified KRP solution (see ESM Methods) for 15 min at 37°C. After appropriate stimulation, [^3^H]2-deoxy-d-glucose (Perkin Elmer, Coventry, UK) was added at 37 kBq/ml for 5 min. Solubilised cell suspensions were collected, and radioactivity was measured in dpm using a multi-purpose scintillator counter (Beckman Coulter, High Wycombe, UK). Each condition was performed in duplicate or triplicate.

### Quantitative RT-PCR

Total RNA was isolated using TRIzol Reagent (Invitrogen/Thermo Fisher), and cDNA was synthesised using a high-capacity RNA-cDNA kit (Applied Biosystems/Thermo Fisher). Quantitative (q)RT-PCR was performed using SYBR green (Sigma) in an Applied Biosystems StepOnePlus system and mRNA normalised to β-actin. Primer sequences are listed in ESM Methods.

### Western blotting and antibodies

Total protein lysates were extracted using RIPA lysis buffer (Sigma), resolved on 7.5–10% SDS-polyacrylamide gels and blotted onto nitrocellulose membranes. Membranes were incubated in primary antibodies overnight at 4°C, before washing and incubation with the appropriate HRP-conjugated secondary antibody (Sigma) at a 1:10,000 dilution. Immunoreactive bands were visualised using Clarity ECL Western Blotting Substrate (Bio-Rad, Hemel Hempstead, UK) on a GE AI600 imager and quantified using ImageJ (NIH, https://imagej.nih.gov/ij/). Primary antibodies are listed in ESM Methods.

### Immunoprecipitation

For immunoprecipitation studies, 5 μg of anti-IRβ (C-19)-AC or Rabbit IgG-AC (Santa Cruz, Dallas, TX, USA) was added directly to cell lysates and incubated overnight at 4°C, under constant rotation. Immune complexes were pelleted at 10,000 *g* for 30 s and washed in lysis buffer. Immune complexes were eluted at 70°C for 10 min. Immunoprecipitation samples were resolved on 7.5% SDS-PAGE gels before western blotting.

### Semi-automated immunofluorescent imaging and analysis

Cells were grown in 96-well plates (Greiner, Stonehouse, UK), stimulated as indicated before fixation and immunostaining. Image acquisition was automated using an IN Cell Analyzer (GE Healthcare, Amersham, UK) imaging platform, and quantification was performed using IN Cell Analyzer work station 3.5 software. Three technical replicates were performed within each experiment, with four fields of view per well, yielding data for around 2000 cells per condition, per experiment. Additional details can be found in ESM Methods.

### Statistical analysis

Data are presented as means ± SEM unless otherwise stated. Statistical analysis was performed using GraphPad Prism (GraphPad Software, La Jolla, CA, USA). Statistical significance was calculated with one-way ANOVA with Tukey’s multiple comparison post hoc analysis, or unpaired two-tailed *t* tests and taken as *p* < 0.05.

## Results

### Culturing insulin-sensitive podocytes in a diabetic environment induces cellular insulin resistance

**Fig. 1:**
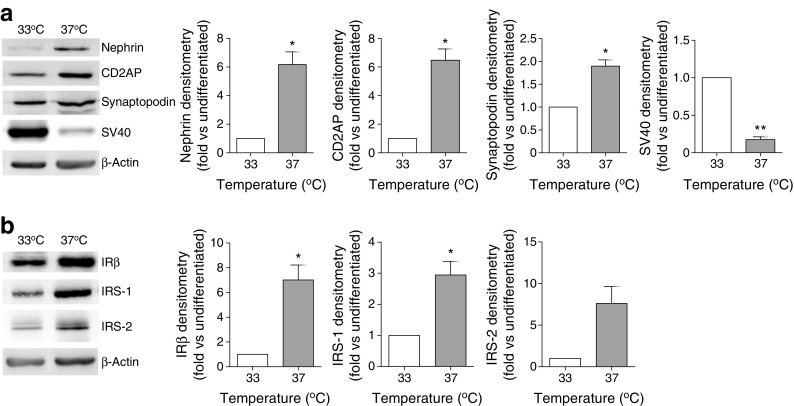
Expression of podocyte markers and key insulin signalling proteins in WT mouse podocytes. Representative western blots and densitometry demonstrating (**a**) levels of the podocyte markers nephrin, CD2-associated protein (CD2AP), synaptopodin and the heat-sensitive SV40 transgene, and (**b**) insulin signalling proteins IRβ, IRS-1 and IRS-2, normalised to β-actin levels, under conditions of proliferation (33°C) and differentiation (37°C). *p < 0.05, **p < 0.01, unpaired *t* test, *n* = 3

Studying conditionally immortalised mouse podocytes [22], we initially demonstrated that, under basal conditions, these cells express key podocyte proteins and components of the insulin signalling cascade, which increase following podocyte differentiation (Fig. 1).

**Fig. 2:**
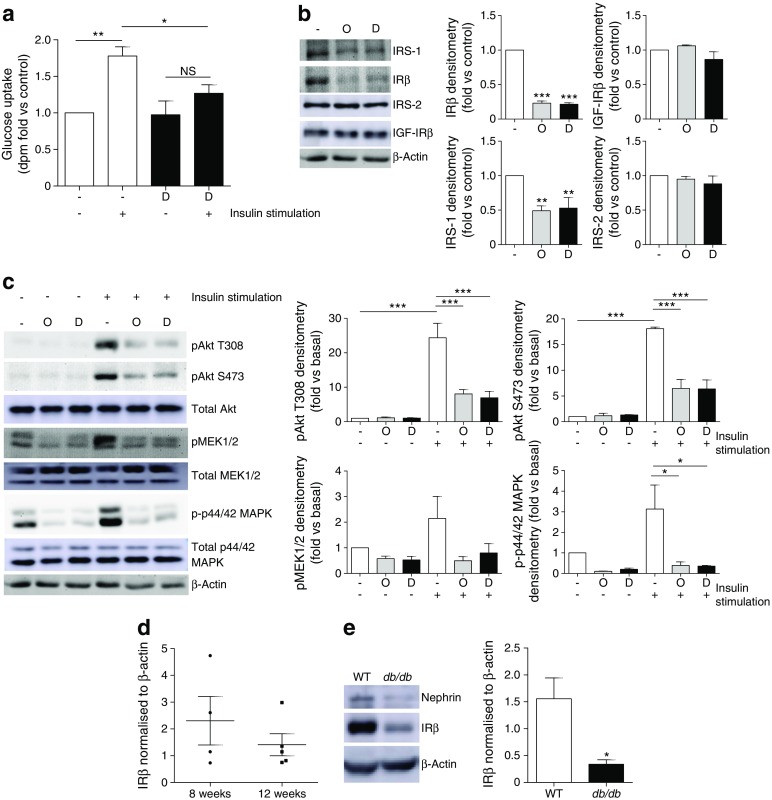
Loss of IRs in diabetic mouse podocytes in vitro and in glomeruli from *db/db* mice. WT mouse podocytes were treated for 10 days with 1 ng/ml TNF-α, 1 ng/ml IL-6, 100 nmol/l insulin and 25 mmol/l glucose (labelled Diabetic, D), prior to insulin stimulation (100 nmol/l). (**a**) dpm counts representing cellular uptake of [^3^H]2-deoxy-d-glucose following exposure of podocytes to the diabetic factors (D); *n* = 4. (**b**) Representative western blots and densitometry of IRS-1, IRβ, IRS-2 and IGF-IRβ protein following exposure of podocytes to the diabetic factors (D), or with mannitol (in parallel with insulin and inflammatory cytokines) included in place of glucose as an osmotic control (O); *n* = 4. (**c**) Representative western blots and densitometry of insulin-stimulated phosphorylation of Akt (T308, S473), mitogen-activated ERK-activating kinase (MEK1/2) and p44/42 MAPK (Thr202/Tyr204); *n* = 3. (**d**) IR protein in glomeruli isolated from *db/db* mice at 8 (*n* = 4, two males and two females) and 12 weeks (*n* = 5, three males and two females) of age. (**e**) IRβ protein in podocyte cultures isolated from 3-month-old *db/db* mice and WT littermate controls; *n* = 6. *p < 0.05, **p < 0.001, ***p < 0.001, one-way ANOVA, Tukey’s multiple comparison

To determine whether these podocytes become insulin resistant following exposure to a diabetic environment in vitro, cells were exposed to common factors associated with insulin resistance—inflammatory cytokines TNF-α and IL-6, high glucose and high insulin—for 10 days. Under these conditions, a significant reduction in insulin-stimulated glucose uptake was observed (Fig. 2a) and, interestingly, a significant reduction in podocyte IR and IRS-1 protein, although IGF-IR and IRS-2 were unchanged (Fig. 2b)*.* These diabetic podocytes also showed a significant reduction in the insulin-stimulated phosphorylation of Akt (S473, T308) and p44/42 mitogen-activated protein kinase (MAPK) (Thr202/Tyr204) (Fig. 2c).

We have recently shown that *db/db* mice on a DBA2/J background develop albuminuric renal disease in correlation with the level of systemic insulin resistance [21]. Glomeruli isolated from these mice demonstrated a trend towards a reduction in IR levels between 8 and 12 weeks of age (as diabetes and albuminuria progresses) (Fig. 2d). To determine podocyte IR production in this model, we isolated podocytes from male *db/db* mice and male WT littermate control animals at 12 weeks of age and generated conditionally immortalised cell lines. Interestingly, podocytes isolated from *db/db* animals had a significantly lower level of IR protein than podocytes isolated from WT littermate control mice (Fig. 2e). Primary podocytes isolated from *db/db* mice also showed reduced IR production when compared with age- and sex-matched control mice (ESM Fig. 1).

Thus, factors associated with systemic insulin resistance modulate podocyte IR levels, attenuating insulin-stimulated phosphorylation cascades.

### Chronic insulin exposure alone is responsible for podocyte IR loss and is sufficient to attenuate podocyte insulin signalling

**Fig. 3:**
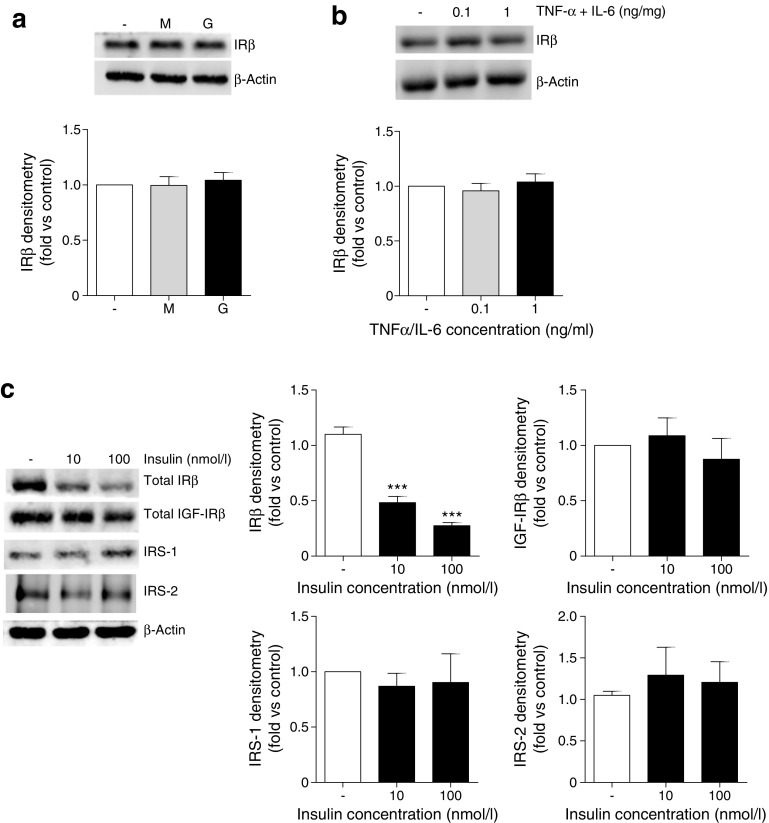
Chronic insulin exposure is responsible for the loss of IR protein in mouse podocytes. Total IRβ levels following the exposure of podocytes to either (**a**) 25 mmol/l glucose (G) or mannitol (M), or (**b**) TNF-α and IL-6, at the stated concentrations, for 10 days. Representative western blot (top) and matched densitometry (bottom). (**c**) Total IRβ, IGF-IRβ, IRS-1 and IRS-2 protein following growth of podocytes in the presence of insulin in vitro. ***p < 0.001, one-way ANOVA, Tukey’s multiple comparison; *n* = 4

We next determined whether any individual factors were responsible for IR loss. Whereas chronic exposure to high glucose or TNF-α and IL-6 in isolation had no significant effect on podocyte IR levels (Fig. 3a, b), chronic exposure to insulin alone caused a significant reduction in podocyte IR protein, while IGF-IR, IRS-1 and IRS-2 were unchanged (Fig. 3c).

**Fig. 4:**
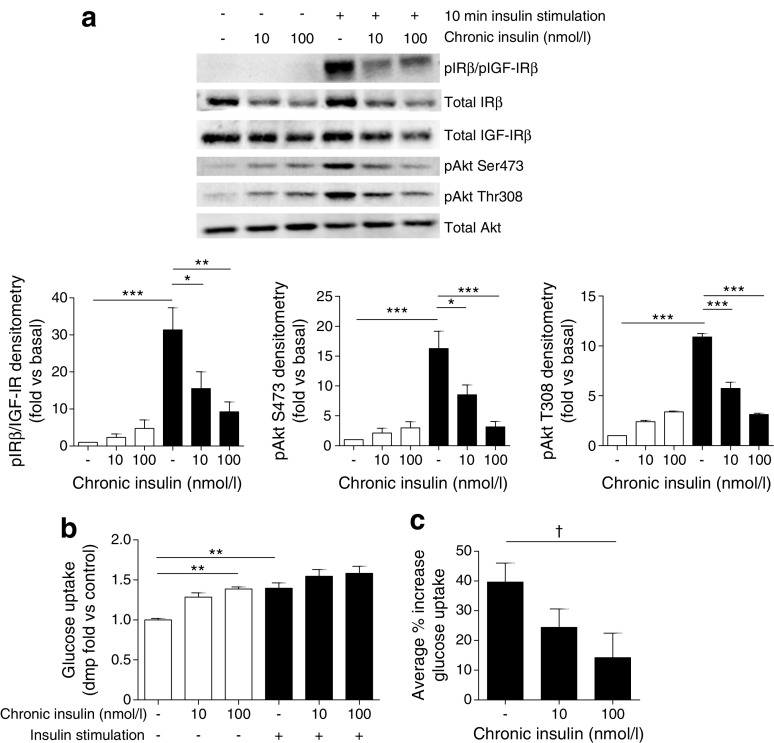
Chronic insulin exposure is sufficient to attenuate podocyte insulin signalling. Mouse podocytes were grown in the presence of insulin (10 and 100 nmol/l) prior to further insulin stimulation (black bars). (**a**) Representative western blots and matched densitometry of insulin-stimulated phosphorylation cascades; *n* = 4. (**b**, **c**) Insulin-stimulated glucose uptake assays; *n* = 3 in duplicate. ^†^ *p* = 0.041, unpaired *t* test; *p < 0.05, **p < 0.01, ***p < 0.001, one-way ANOVA, Tukey’s multiple comparison

Consistent with reduced IR protein, we also found that chronic insulin exposure was sufficient to attenuate insulin-stimulated phosphorylation of IR/IGF-IRs (using a phospho-specific antibody recognising both IR and IGF-IR) and Akt in podocytes. This was accompanied by a reduction in insulin-stimulated glucose uptake (Fig. 4).

**Fig. 5:**
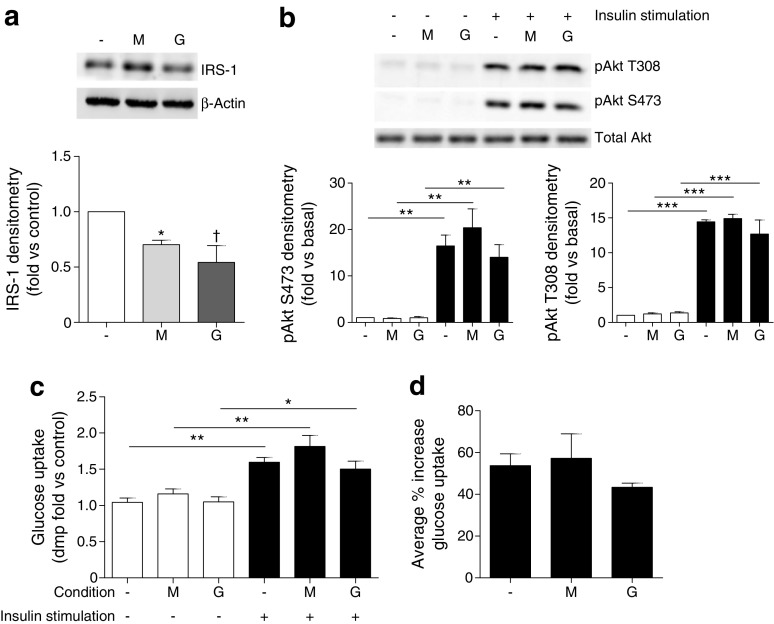
High glucose is the cause of IRS-1 loss in podocytes. Mouse podocytes were grown in the presence of 25 mmol/l glucose (G) or mannitol (M) for 10 days prior to insulin stimulation (100 nmol/l, 10 min, black bars). (**a**) Representative western blot and densitometry of IRS-1 protein in podocytes. *n* = 3; *p < 0.05; ^†^ *p* = 0.09 vs control cells. (**b**) Representative western blots and densitometry of insulin-stimulated signalling in podocytes following chronic glucose exposure; *n* = 3. (**c, d**) Insulin-stimulated glucose uptake assays (*n* = 4), in duplicate. *p < 0.05, **p < 0.01, ***p < 0.001; no significant difference between any of the insulin-stimulated groups, one-way ANOVA, Tukey’s multiple comparison

As podocyte IRS-1 was decreased in diabetic conditions (Fig. 2), we also examined the factors influencing IRS-1 levels. In contrast to the IR, chronic exposure to elevated glucose concentrations alone caused a significant reduction in podocyte IRS-1, as opposed to chronic insulin or inflammatory cytokine exposure (Fig. 5a). There was, however, no significant attenuation in insulin-stimulated phosphorylation cascades or glucose uptake under these conditions (Fig. 5b–d).

### Increased IR degradation is responsible for insulin-induced IR loss in podocytes

**Fig. 6:**
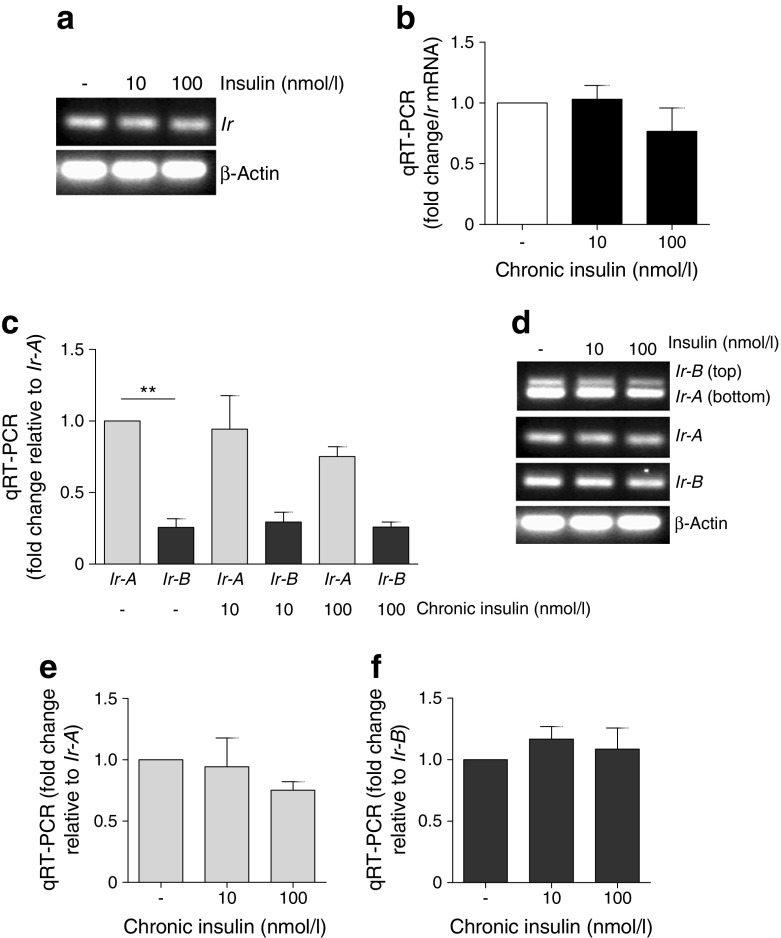
No significant changes in *Ir* mRNA in podocytes following chronic insulin exposure. (**a**) Endpoint RT-PCR and (**b**) qRT-PCR of *Ir* mRNA following chronic exposure of mouse podocytes to insulin; *n* = 4. (**c**) Representative qRT-PCR and (**d**) endpoint RT-PCR demonstrating the relative abundance of podocyte *Ir-A/B* mRNA following growth in chronic insulin. **p < 0.01. Individual changes in (**e**) *Ir-A* and (**f**) *Ir-B* mRNA relative to basal condition. No significant differences, unpaired *t* test; *n* = 4 in triplicate

To elucidate the mechanisms underlying IR loss in diabetic podocytes, we initially investigated whether insulin had any effect on IR transcription. Although insulin-induced inhibition of *IR* (also known as *INSR*) mRNA has been reported in other cell systems [26, 27], chronic insulin had no significant effect on mouse podocyte *Ir* mRNA levels (Fig. 6a, b).

The IR also exists as two isoforms as a consequence of the differential splicing of exon 11 [32], and the relative abundance of IR-A/B has been linked to hyperinsulinaemia [33]. As podocytes produce both IR isoforms [34], we quantified the relative abundance of *Ir-A/B* mRNA in podocytes using isoform-specific primers [35]. Although a significantly higher proportion of *Ir-A* mRNA (than that encoding *Ir-B*) was observed in podocytes, there was no significant effect of chronic insulin exposure on the relative abundance of either isoform (Fig. 6c–f).

**Fig. 7:**
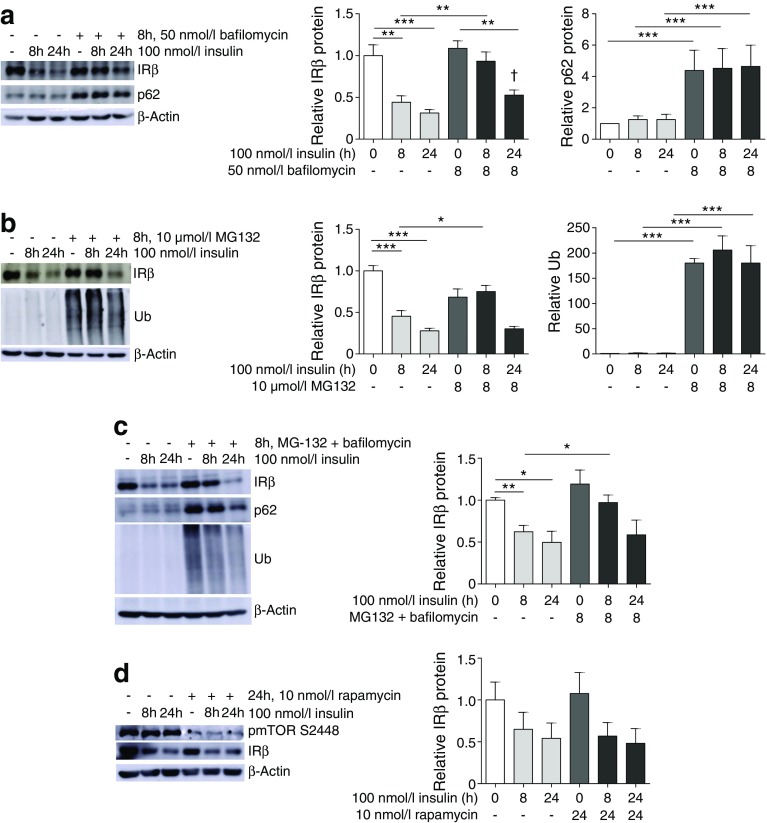
Proteasome and lysosome inhibition blocks insulin-induced IR degradation in podocytes. Podocytes were treated with (**a**) 50 nmol/l bafilomycin, (**b**) 10 μmol/l MG132, or (**c**) both bafilomycin and MG132 for 8 h, alone or in combination with insulin for 8 or 24 h. IRβ levels were determined by western blotting. Levels of p62 and ubiquitin (Ub) were determined as positive controls for successful lysosomal and proteasomal inhibition, respectively. (**d**) Representative western blots and matched densitometry showing IRβ levels after mTOR inhibition (24 h, 10 nmol/l rapamycin) alone or in combination with chronic insulin stimulation; phosphorylation of mTOR (S2448) was determined as a positive control for successful mTOR inhibition; *n* = 4–6 experiments. *p < 0.05, **p < 0.01, ***p < 0.001, one-way ANOVA, Tukey’s multiple comparison; ^†^ *p* = 0.0151, two-tailed *t* test vs 24 h

The role of post-translational degradation on insulin-induced IR loss in podocytes was next examined. Consistent with the above results, chronic insulin stimulation caused a significant reduction in the levels of IR protein. Inhibition of lysosomal degradation in podocytes with bafilomycin (confirmed by a significant increase in p62, a protein typically degraded via autophagy and lysosomal pathways [36]) reduced this insulin-induced IR loss (Fig. 7a).

Inhibition of proteasomal degradation in podocytes by an 8 h incubation with the 26S proteasome inhibitor MG132 (confirmed by an increase in ubiquitinated proteins) also delayed insulin-induced IR loss (Fig. 7b). In addition, we found evidence of IR ubiquitination. A small increase in ubiquitination was observed in IRβ immunoprecipitates where mouse podocytes were treated with both insulin and MG132. In mouse podocytes overproducing IR, we observed strong ubiquitin signals, confirming these results (ESM Fig. 2).

We saw no additional effect where proteasome and lysosome inhibitors were used in combination (Fig. 7c).

As mechanistic target of rapamycin (mTOR) is activated by insulin signalling [37], and may also regulate autophagy [38], we also investigated whether mTOR signalling had any involvement in insulin-induced insulin resistance in podocytes. Inhibition of mTOR with rapamycin had no significant effect on IR levels (Fig. 7d).

### Restoring insulin signalling in insulin-resistant human podocytes requires stable expression of both IR and nephrin

Human podocytes used in previous studies [15, 39, 40] have become ‘naturally’ insulin resistant as a consequence of continuous and historical cell culture conditions, whereby cell culture media were supplemented with insulin at concentrations in excess of 200 nmol/l [24]. To determine whether IR loss was a primary cause of insulin resistance in these cells, human podocytes (WT) were stably transfected with the human IR using lentivirus (WT-IR podocytes).

**Fig. 8:**
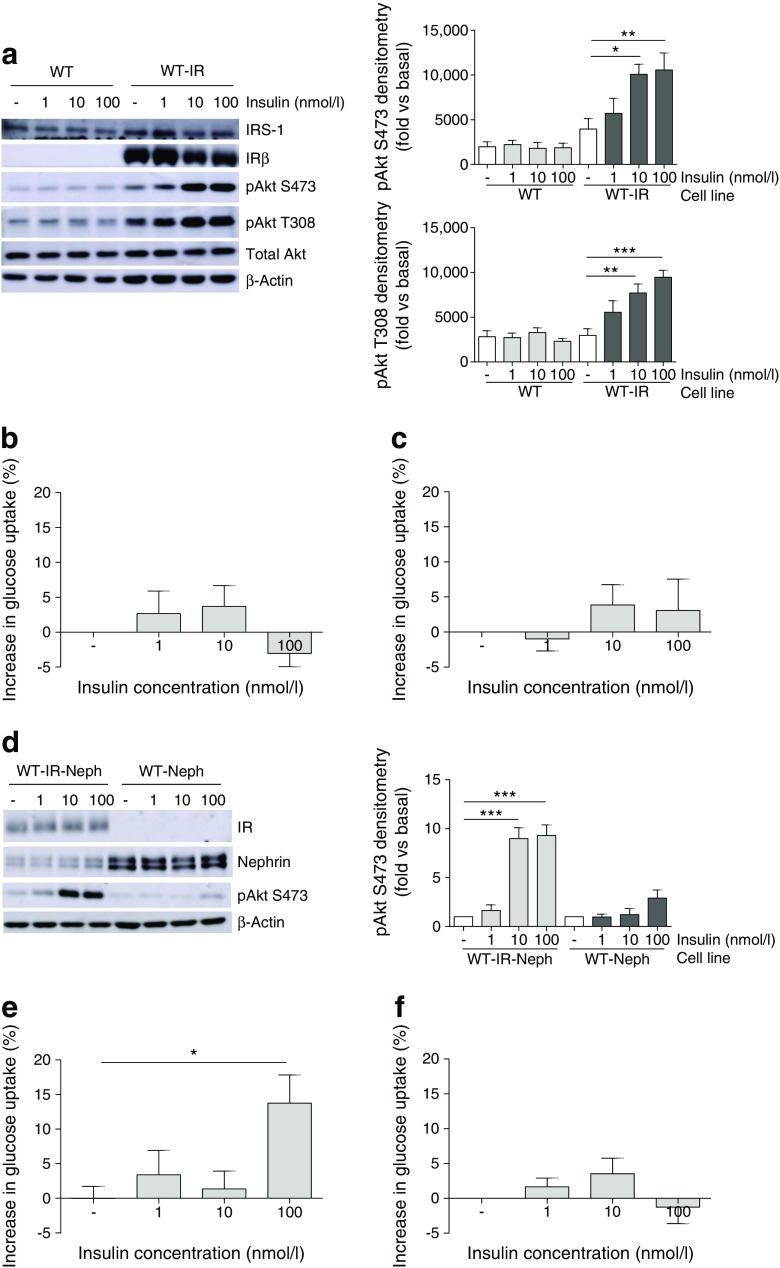
Stable IR and nephrin expression rescues insulin signalling in insulin-resistant human podocytes. Human (WT) podocytes were stably transfected with lentiviral particles containing human IR (WT-IR), human nephrin (WT-Neph) or both (WT-IR-Neph), and insulin responses were investigated. (**a**) Representative western blots and matched densitometry demonstrating increased phosphorylation of Akt (S473, T308) in WT-IR podocytes. *p < 0.05, **p < 0.01, ***p < 0.001, one-way ANOVA, Tukey’s multiple comparison; *n* = 4. Insulin-stimulated glucose uptake experiments showed no significant increase in this response in either (**b**) WT or (**c**) WT-IR podocytes. (**d**) Representative western blots demonstrating stable expression of nephrin and IR in appropriate cell lines, and insulin-stimulated phosphorylation (nmol/l, 10 min) of Akt (S473); *n* = 3. (**e**) WT-IR-Neph cells showed a significantly increased percentage glucose uptake following insulin stimulation (100 nmol/l), compared with unstimulated cells. *p = 0.0142 Mann–Whitney vs unstimulated cells. (**f**) WT-Neph cells have no significant increase in insulin-stimulated glucose uptake; *n* = 4

In contrast to WT podocytes, there was a significant increase in phosphorylation of Akt following insulin stimulation of WT-IR podocytes (Fig. 8a). However, there was no significant increase in the glucose uptake response in either cell line (Fig. 8b).

As we have previously demonstrated that nephrin is necessary for insulin-stimulated glucose uptake in podocytes [39, 40], and nephrin loss is also evident in diabetic environments [41–43], the effect of stable nephrin overproduction in insulin-resistant human podocytes was investigated. As shown in Fig. 8d, podocytes stably producing both IR and nephrin (WT-IR-Neph) retained their insulin-stimulated phosphorylation of Akt. In addition, a significant increase in glucose uptake into WT-IR-Neph podocytes was observed (Fig. 8e). Overproduction of nephrin alone did not rescue insulin-stimulated Akt phosphorylation or glucose uptake (Fig. 8d, f).

As nephrin has a key role in regulating the podocyte actin cytoskeleton [44], and the insulin-stimulated trafficking of glucose transporters (in GLUT storage vesicles) to the plasma membrane relies on the actin cytoskeleton [45], we hypothesised that the requirement of nephrin for glucose uptake in podocytes might also be related to actin modulation. As such, we next investigated insulin-stimulated actin reorganisation in these cells, using automated microscopy and image analysis. Cells positive for actin remodelling were defined as those with a loss of defined (central) F-actin structures, as this is generally considered to indicate reorganisation [46].

**Fig. 9:**
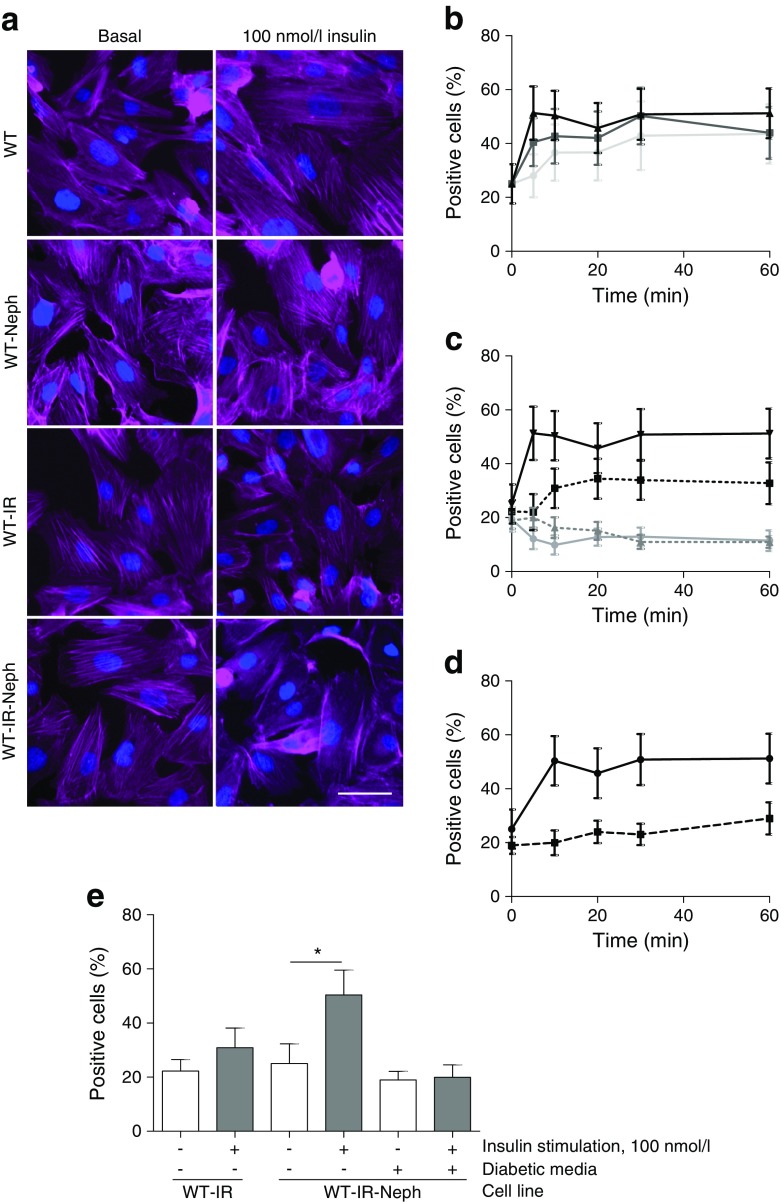
IR and nephrin expression are both required for effective insulin-stimulated actin remodelling in podocytes. Human (WT) podocytes were stably transfected with lentiviral particles containing human nephrin (WT-Neph), human IR (WT-IR) or both (WT-IR-Neph). Cell lines were stimulated with insulin at the stated doses and times, prior to F-actin and nuclear staining. The percentage of cells positive for actin reorganisation was calculated as described in the Methods. Modest changes in brightness and contrast were uniformly applied to all images for visual purposes; unmodified images were used for quantification. (**a**) Representative fluorescent images of each cell line under basal conditions and following insulin stimulation (100 nmol/l, 10 min). Scale bar, 50 μm. (**b**) Percentage of WT-IR-Neph cells displaying evidence of F-actin remodelling when stimulated with insulin at 1 nmol/l (light grey line), 10 nmol/l (dark grey line) and 100 nmol/l (black line). (**c**) Comparison of responses between stable cell lines (grey line, WT; grey dotted line, WT-Neph; black dotted line, WT-IR; black line, WT-IR-Neph) stimulated with 100 nmol/l insulin over the time course. (**d**) Effect of insulin-resistant conditions on actin remodelling in WT-IR-Neph cells stimulated with 100 nmol/l insulin over the time course (black line, basal conditions; black dotted line, diabetic media). (**e**) Bar graph of data from (**c**, **d**) for 100 nmol/l insulin at 10 min. A significant increase in percentage of WT-IR-Neph podocytes positive for actin remodelling was observed; this response was lost in WT-IR-Neph podocytes exposed to insulin-resistant conditions. *p = 0.036 Mann–Whitney test, *n* = 3

Representative images of podocytes stimulated with 100 nmol/l of insulin for 10 min are presented in Fig. 9a, which were quantified using an IN Cell Analyzer. In WT-IR-Neph podocytes, insulin caused a rapid dose-dependent increase in F-actin remodelling (Fig. 9b). In contrast, insulin stimulation had no effect on actin reorganisation in WT cells or WT cells producing nephrin (WT-Neph), and only a modest effect in WT-IR podocytes (Fig. 9c, e). We also found that the actin remodelling observed in WT-IR-Neph podocytes was completely blocked when these cells were exposed to a diabetic environment (Fig. 9d, e).

## Discussion

Hyperinsulinaemia is a major metabolic abnormality associated with systemic insulin resistance [47]. In addition to the association between insulin resistance and albuminuria [4–6], hyperinsulinaemia per se is related to albuminuria in type 2 diabetes [48] and in type 1 diabetes, where nephropathy is associated with higher therapeutic doses of insulin [49].

At a cellular level, hyperinsulinaemia may exacerbate insulin resistance, disrupting insulin action at several points within the signalling cascade, in a cell-specific manner [50]. At the level of the IR, chronic insulin stimulation can promote receptor downregulation in classically insulin-responsive tissues such as skeletal muscle and liver [26–28], as well as other cell types including pancreatic alpha cells [25] and cultured neurones [29, 30].

The present study demonstrates, for the first time, that the glomerular podocyte is also subject to insulin-induced IR degradation, resulting in an attenuation of insulin signalling responses. Given that we [16] and others [17] have shown the podocyte-specific knockdown of the IR induces albuminuria and glomerular disease, it is reasonable to predict that this insulin-induced IR loss could further contribute to renal disease in settings of diabetes and insulin resistance.

Mechanistically, we demonstrated that insulin-induced IR degradation occurs via a proteasome-dependent and bafilomycin-sensitive pathway. In contrast to studies performed in other cell systems [26, 27], we found that chronic insulin stimulation of podocytes had no significant effect on IR mRNA. We did, however, demonstrate that the relative abundance of IR-A mRNA is greater than that of IR-B in podocytes. As IR-A is the isoform thought to be responsible for IR-mediated IGF-II signalling, this may be of relevance in IGF-II signalling to this cell [32, 51].

Although we found that the insulin-induced degradation of IRS-1 [29] and IRS-2 [52] observed in other cell systems does not occur in podocytes, in agreement with previous studies performed in a rat model of streptozotocin-induced diabetes and glomerular endothelial cells [53], we detected a reduction in podocyte IRS-1 content as a consequence of high glucose exposure. Our finding that there was no significant reduction in the insulin-stimulated phosphorylation of Akt or glucose uptake under these conditions is in line with our recent studies suggesting that IRS-2, as opposed to IRS-1, is the principal isoform facilitating podocyte insulin signal transduction to stimulate Akt phosphorylation and glucose uptake [54]. However, this does not eliminate the possibility that IRS-1 could mediate other insulin responses in podocytes, or selectively modulate individual Akt isoforms.

We also demonstrate that while initial insulin signalling events leading to Akt phosphorylation in podocytes do not require nephrin, the stable expression of both IR and nephrin is necessary to mediate insulin-stimulated glucose transport downstream of Akt. Interestingly, it is well documented that nephrin production is also lost early in human diabetic nephropathy [43] and experimental models of diabetes [41, 42].

We hypothesised, given the importance of actin regulation in the insulin-stimulated glucose uptake response [45], that the requirement for nephrin in glucose uptake might also be linked to the role of nephrin in modulating the podocyte actin cytoskeleton [44], a process that is closely related to podocyte function at the filtration barrier. As such, we investigated how insulin-stimulated actin remodelling was affected in our model systems, and found that podocytes with stable over expression of both nephrin and IR were more responsive to insulin in terms of F-actin remodelling, compared with podocytes overexpressing IR alone.

Others have shown nephrin may also interact with the IR [34], investigating whether nephrin also affects localisation of IR, or indeed IR binding to downstream targets, may be of interest. Whether this may also be linked to the ability of nephrin to preferentially phosphorylate selective Akt isoforms remains to be investigated, as Akt2 has been suggested to differentially modulate the podocyte actin cytoskeleton in comparison to Akt1 [55]. In addition, we found that the actin remodelling downstream of insulin was blocked following exposure of these podocytes to a diabetic environment, suggesting that these cells may still become insulin resistant following further challenge with a diabetic environment.

Aside from enhancing cellular glucose uptake and promoting the reorganisation of F-actin structures, podocyte IR signalling has the capacity to regulate vascular endothelial growth factor (VEGF)-A production [56], cell viability [20], reactive oxygen species (ROS) production and autophagy [57], all of which are key in maintaining glomerular filtration and are dysregulated in diabetes. Recently, the importance of IR signalling in the adaptation of podocytes to endoplasmic reticulum stress and the unfolded protein response in diabetes has further highlighted the physiological importance of this pathway in disease responses [17]. A further study has also linked podocyte IR signalling to mitochondrial function, which may be related to the modulation of mTOR signalling [58], which is also crucial in the maintenance of podocytes in health and disease.

**Fig. 10:**
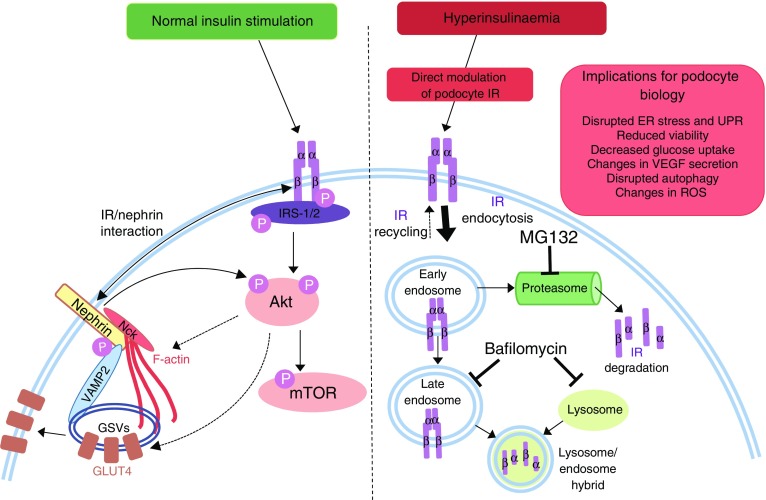
Proposed mechanism of podocyte IR degradation and its consequences. Normal insulin signalling to podocytes leads to phosphorylation of IRS proteins, predominantly IRS-2 [54], and activation of downstream signalling events, including the phosphorylation of Akt and mTOR. Nephrin is also required for insulin-stimulated glucose uptake (in part via interaction of nephrin with VAMP2 [39, 40] and insulin-stimulated actin remodelling). Hyperinsulinaemia, occurring in diabetes and insulin resistance, causes an increase in proteasomal and lysosomal degradation of the podocyte IR, attenuating downstream signal transduction. Ultimately, loss of podocyte IRs may exacerbate albuminuria and features of diabetic nephropathy, dysregulating the ER stress response [17], VEGF-A secretion [56], ROS production [57], autophagy [57], cell viability [20] and actin remodelling, and reducing glucose uptake. ER, endoplasmic reticulum; GSV, GLUT storage vesicle; UPR, unfolded protein response; VAMP2, vesicle-associated membrane protein 2

As insulin has the capacity to modulate a number of factors vital in podocyte function, we propose that hyperinsulinaemia, via a direct regulation of podocyte IR levels, may directly influence podocyte biology and, subsequently, glomerular function, as depicted in Fig. 10.

In summary, this study demonstrates that podocytes exposed to a diabetic environment become insulin resistant. This response is, at least in part, mediated by chronic exposure to elevated insulin levels promoting podocyte IR degradation. Moreover, we demonstrated that the expression of both IR and nephrin is required for full podocyte insulin sensitivity. Thus, hyperinsulinaemia may further accentuate the progression of renal disease in states of insulin resistance, including diabetic nephropathy, and the maintenance of podocyte nephrin and IR expression and function in these settings may be therapeutically beneficial.

## Electronic supplementary material

ESM (PDF 349 kb)

## Abbreviations

IR: Insulin receptor
MAPK: Mitogen-activated protein kinase
mTOR: Mechanistic target of rapamycin
qRT-PCR: Quantitative RT-PCR
ROS: Reactive oxygen species
WT: Wild type
WT-IR-Neph: Wild-type cells producing insulin receptor and nephrin
WT-Neph: Wild-type cells producing nephrin
VEGF: Vascular endothelial growth factor
