# Supplementary material for: Prolonged exposure of mouse and human podocytes to insulin induces insulin resistance through lysosomal and proteasomal degradation of the insulin receptor
Source: Diabetologia. 2017 Aug 29;60(11):2299–311. doi: 10.1007/s00125-017-4394-0 (PMC6448913; doi:10.1007/s00125-017-4394-0)
Supplement: Supplementary file 1 — (PDF 349 kb) [file 125_2017_4394_MOESM1_ESM.pdf]

# Supplementary Material

## ESM Methods

**Animals** All animal experiments and procedures were approved by the UK Home Office in accordance with the Animals (Scientific Procedures) Act 1986 and the Guide for the Care and Use of Laboratory Animals was followed during experiments. Heterozygous db/wt DBA/2J (D2.BKS(D)-*Lepr*$^{db}$/J) mice were purchased from The Jackson Laboratory (Bar Harbor, US). Female and male *db/db* mice were bred in house, as described [1].

All mice were housed in a 12/12 h light/dark cycle with free access to standard chow (EURodent Diet 22%, percentage of energy: protein 25.9%, fat 9.3%, carbohydrate 64.8%; LabDiet, 104 St. Louis, MO, US) and water. We have previously reported the renal phenotype of these mice and that the urinary albumin creatinine in these animals correlates closely with systemic insulin resistance [1]. As reported, by 12 weeks of age *db/db* animals are hyperglycaemic, hyperinsulinaemic with a significantly elevated body weight compared to wild-type littermate controls. ACRs and insulin resistance also progress between 8 and 12 weeks of age [1].

**Isolation of mouse glomeruli** Glomeruli were isolated from db/db mice using dynabead perfusion, as previously reported [2]. Briefly, mice were anaesthetised by intraperitoneal injection of euthatal and perfused through the heart with Hank's balanced salt solution (HBSS) before dynabead perfusion (8 x $10^7$ diluted in HBSS). Kidneys were removed and digested in 1mg/ml collagenase A, before passage through a 100µm cell strainer and pelleted at 200g for 5 minutes. Glomeruli were isolated from digested kidney tissue using a magnetic concentrator, for protein analysis or podocyte isolation and generation of conditionally immortalised cell lines.

**Glucose uptake** For glucose uptake experiments, modified KRP solution consisted of the following; Ca-free KREBS salts, sodium phosphate, 1.25mM $CaCl_2$, 2mM NaHCO3, 25mM HEPES. Radiolabelled glucose was purchased from Perkin Elmer (DEOXY-D-GLUCOSE, 2-[1,2-3H (N)] #NET328A250UC). A final concentration of 50µM 3H-deoxy-D-glucose was added to stimulated podocytes (equivalent to 1µCi/ml).

**Quantitative-Polymerase Chain Reaction**

Primer sequences were purchased from Eurofins Scientific UK and as follows:

| Target | Sequence | Product length |
|---|---|---|
| Mouse *IR-A** | F: 5’ TCCTGAAGGAGCTGGAGGAGT 3’ | 89bp |
| | R: 5’CTTTCGGGATGGCCTGG 3’ | |
| Mouse *IR-B** | F: 5’ TCCTGAAGGAGCTGGAGGAGT 3’ | 123bp |
| | R: 5’ TTCGGGATGGCCTACTGTC 3’ | |
| Mouse Total *IR* | F: 5’ GCTTCTGCCAAGACCTTCAC 3’ | 94bp |
| | R: 5’ CACTCGGGGATGCACTTATT 3’ | |
| *β-Actin* | F: 5’ GACAGGATGCAGAAGGAGATTACT 3’ | 142bp |
| | R: 5’ TGATCCACATCTGCTGGAAGGT 3’ | |

**IR-A* and *IR-B* primers for comparative qRT-PCR were reported previously and have matched amplification efficiencies [3].

**Western blotting antibodies** Primary antibodies against Total Akt, phospho-Akt (S473, T308), Phospho-IGF-IRβ (Tyr1135/1136)/IRβ (Tyr1150/1151), Total IRβ, Total IGF-IRβ, IRS-1, IRS-2, p-p44/42 MAPK (Thr202/Tyr204), Ubiquitin and p62 were purchased from Cell Signalling technologies and diluted 1:1000. Anti-Nephrin was purchased from Acris and diluted 1:500. β-actin was purchased from Sigma and diluted 1:10,000.

**Immunofluorescent staining and imaging** For automated imaging of cytoskeletal changes, podocytes were grown in 96-well plates (Greiner), stimulated as indicated before fixation in 4% PFA. Cells were then permeabilised in 0.1% Triton-X100 and f-actin structures were visualised using alexafluor phalloidin 647 (Invitrogen). Nuclear regions were defined by Hoechst 33342 stains. Image acquisition was automated using an IN Cell Analyzer (GE Healthcare) high content imaging platform with a 10x objective. Quantification of IN Cell images was performed using the IN Cell Analyser work station 3.5 software. The average fluorescence intensity of the phalloidin staining in the central (nuclear) region (expressed as arbitrary fluorescence units, AFU) was used for quantification. Cells positive for actin remodelling were defined as those with a loss of defined central F-actin structures; as this is generally considered to indicate reorganisation [4]. 3 technical replicates were performed within each experiment, with 4 fields of view per well; yielding data for around 2000 cells per condition, per experiment.

## ESM Figures

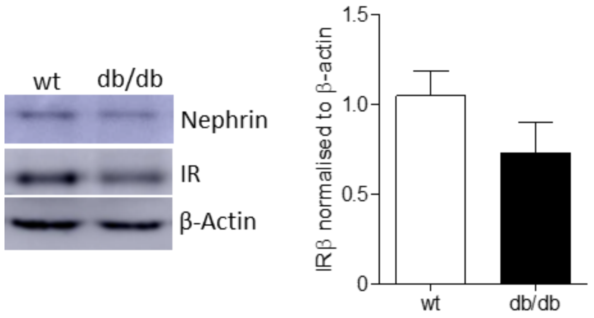


**ESM Fig. 1** Loss of IR expression in primary *db/db* podocytes. Podocytes were isolated from perfused glomeruli of wt and *db/db* age and sex-matched mice and IR protein was determined by western blotting, n=1 animal in triplicate.

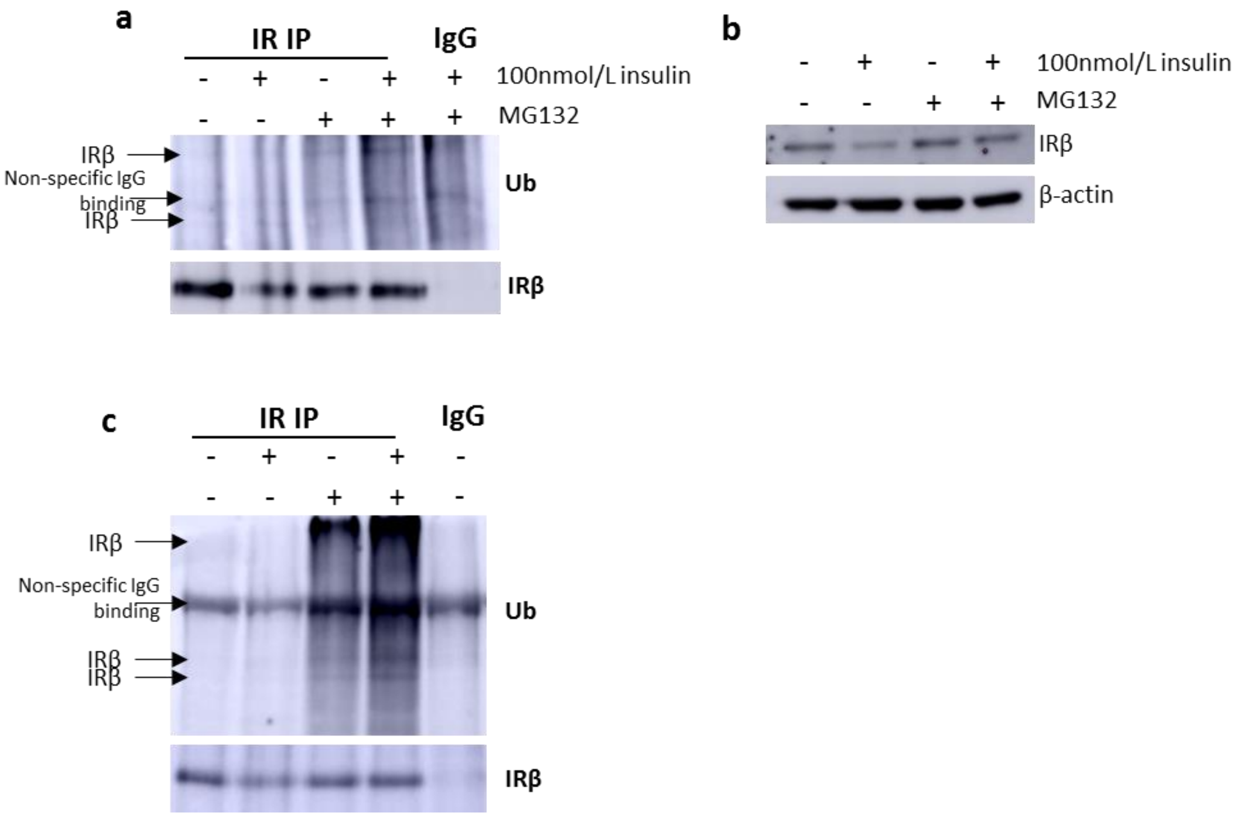


**ESM Fig. 2** Increased ubiquitination of the IR following high insulin exposure. Mouse podocytes were treated with 10µmol/L MG132 for 8 hours alone or in combination with insulin (100nmol/L), before protein extraction and IR immunoprecipitation (IR IP). Levels of Ubiquitin (Ub) and IRβ were determine by western blotting. (**a**) Ubiquitin blots of IR IP samples of wild-type mouse podocytes, which were stripped and re-probed for total IRβ (**b**) Total protein lysates (collected prior to IR IP) confirming co-incubation of podocytes with MG-132 blocks IR loss (**c**) Ubiquitin blots of mouse podocytes over-expressing the IR following IR IP, representative of n=4 experiments.

## References

[1] Ostergaard MV, Pinto V, Stevenson K, Worm J, Fink LN, Coward RJ (2016) DBA2J db/db mice are susceptible to early albuminuria and glomerulosclerosis that correlates with systemic insulin resistance. American journal of physiology Renal physiology: ajprenal.00451.02016
[2] Takemoto M, Asker N, Gerhardt H, et al. (2002) A new method for large scale isolation of kidney glomeruli from mice. The American journal of pathology 161: 799-805
[3] Rowzee AM, Ludwig DL, Wood TL (2009) Insulin-like growth factor type 1 receptor and insulin receptor isoform expression and signaling in mammary epithelial cells. Endocrinology 150: 3611-3619
[4] Tojkander S, Gateva G, Lappalainen P (2012) Actin stress fibers--assembly, dynamics and biological roles. Journal of Cell Science 125: 1855-1864
